# 191. High-Dose Rifampin-containing Regimens for the Treatment of TB Meningitis

**DOI:** 10.1093/ofid/ofab466.191

**Published:** 2021-12-04

**Authors:** Camilo A Ruiz-Bedoya, Filipa Mota, Elizabeth Tucker, Farina Mahmud, Clara Erice, Melissa Bahr, Kelly Flavahan, Patricia De Jesus, John Kim, Marina Da Costa Rosa, Alex Shimura Yamashita, Catherine A Foss, Charles A Peloquin, Charles A Peloquin, Alvaro A Ordonez, Sanjay K Jain, Sanjay K Jain

**Affiliations:** 1 Johns Hopkins University, School of Medicine, Baltimore, MD; 2 Johns Hopkins, Baltimore, Maryland; 3 University of Florida, Gainesville, FL

## Abstract

**Background:**

TB meningitis is the most severe form of tuberculosis (TB), associated with high morbidity and mortality. High-dose rifampin (35mg/kg/day) is safe in adults and substantially improves the bactericidal activity of standard TB regimen. However, there is conflicting data regarding its benefit in TB meningitis where outcomes may also be associated with intracerebral inflammatory responses.

**Methods:**

A novel mouse and a validated rabbit model of TB meningitis utilizing intracranial *Mycobacterium tuberculosis* infections were used for these studies (**Fig. 1**). Animals received high-dose (35 mg/kg/day) or standard-dose (10 mg/kg/day) rifampin in combination with isoniazid, pyrazinamide and dexamethasone at human equipotent dosing. Bacterial burden, multi-modality positron emission tomography (PET) imaging, tissue drug concentrations, markers of neuroinflammation, and vascular leak were measured. Imaging data from a patient with TB meningitis was analyzed and correlated with the findings in animals.

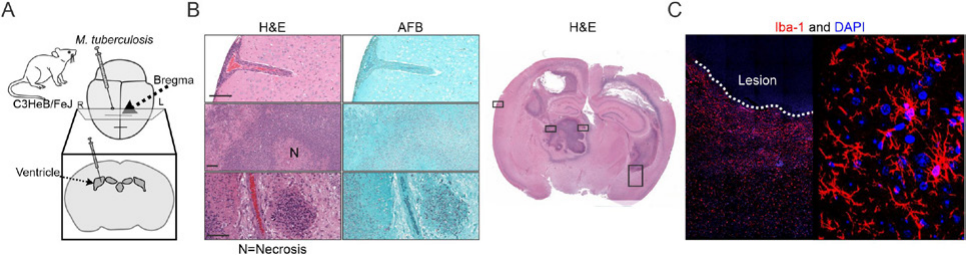

Figure 1. Mouse model of TB meningitis replicates human histopathology hallmarks. (A) Scheme of infection. (B) Histopathology hematoxylin-eosin (H&E) and acid-fast bacilli (AFB) staining in a representative M. tuberculosis-infected mouse shows regions of meningitis, ventriculitis, choroiditis, necrotizing and non-necrotizing granulomas. The bar represents 100µm. (C) Images show immunofluorescence of microglia activation in red (Iba-1) and nuclear stain in blue (DAPI). The rabbit model of TB meningitis has been described previously (Tucker et al. Dis Model Mech. 2016 and Tucker et al. Sci Transl Med. 2018). Animal studies were approved by the Johns Hopkins Animal Care and Use Committee.

**Results:**

Administration of the high-dose rifampin regimen achieved four times higher brain concentration than the standard-dose regimen and displayed higher bactericidal activity in both mice and rabbits (*P* < 0.01) (**Fig. 2**). There were no differences in intracerebral microglial activation (^124^I-DPA-713 PET and iDISCO) and pro-inflammatory cytokines during treatment in animals receiving high- or standard-dose rifampin regimens (**Fig. 3**). Whole-brain PET and immunolabeling demonstrated spatially compartmentalized inflammation, vascular leak and rifampin exposures (**Fig. 4**). Longitudinal imaging in the same animals showed a 40% decrease in vascular leak after two weeks of TB treatment. Spatially compartmentalized brain rifampin exposures and decreases in vascular edema over TB treatment were also noted in the TB meningitis patient.

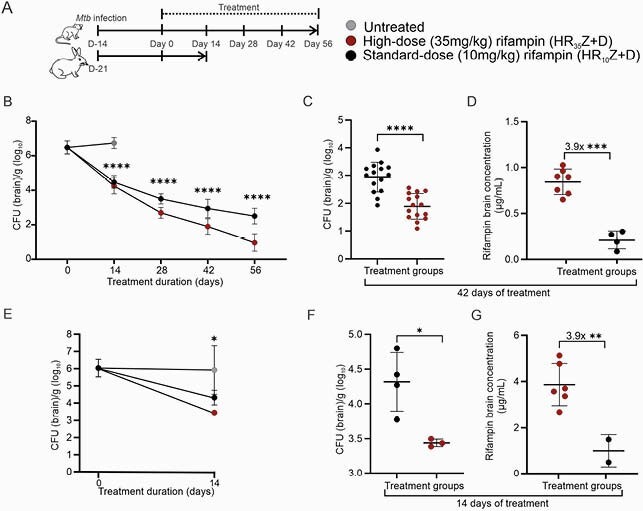

Figure 2. High-dose rifampin treatment in mouse and rabbit models of TB meningitis. (A) Experimental scheme. R10 (rifampin 10mg/kg), R35 (rifampin 35mg/kg), H (isoniazid), Z (pyrazinamide), D (dexamethasone). (B) Bactericidal activity of high-dose rifampin (n = 60 animals) and standard-dose rifampin (n = 60 animals) regimens in mice. (C) Grouped colony forming units (CFU) and (D) rifampin brain concentration in mice after 42 days of TB treatments. (E) Bactericidal activity of high-dose rifampin (n = 4 animals) and standard-dose rifampin (n = 3 animals) regimens in rabbits. Data from untreated rabbits (n = 2 animals) is also shown. (F) Grouped CFU and (G) rifampin brain concentration in rabbits after 14 days of TB treatments. Tissues were assayed using validated ultra-high-performance liquid chromatography and tandem mass spectrometry (LC-MS/MS) for rifampin at the Infectious Diseases Pharmacokinetics Laboratory of the University of Florida. The bacterial burden is represented as CFU per gram of tissue and presented on a logarithmic scale. CFU and mass spectrometry data are represented as mean ± SD. *P < 0.05, **P < 0.01 and ***P < 0.001 calculated using a two-way ANOVA test.

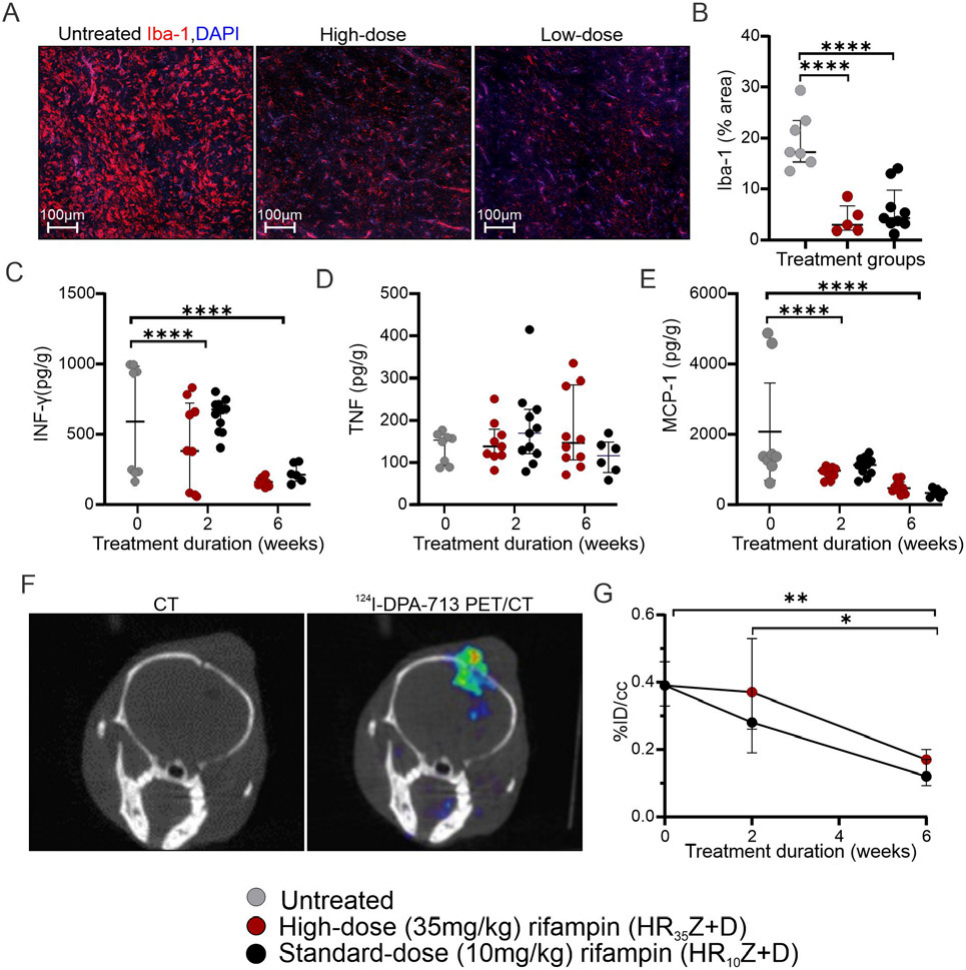

Figure 3. Neuroinflammatory responses during TB treatment. (A) Iba-1 and DAPI immunofluorescence in a representative untreated, high-dose and low-dose-treated mouse. (B) Quantification of Iba-1 immunofluorescence before treatment and after 6 weeks of treatment (n = 3 mice per group). Sections were imaged at 40X with Nikon A1+ confocal microscope. HALO was used for visualization and quantification. Quantification of intraparenchymal (C) INF-γ, (D) TNF and (E) MCP-1 in untreated and treated mice (Milliplex Multiplex Luminex assay). (F) Coronal CT and 124I-DPA-713 PET/CT of a representative mouse with TB meningitis before treatment initiation. (G) 124I-DPA-713 PET quantification before treatment (n = 15 animals) and after 2 (n = 5 animals per group) and 6 (n = 5 animals per group) weeks of TB treatment. PET data is represented as median ± IQ. *P < 0.05, **P < 0.01 and ***P < 0.001 were calculated using a two-way ANOVA test.

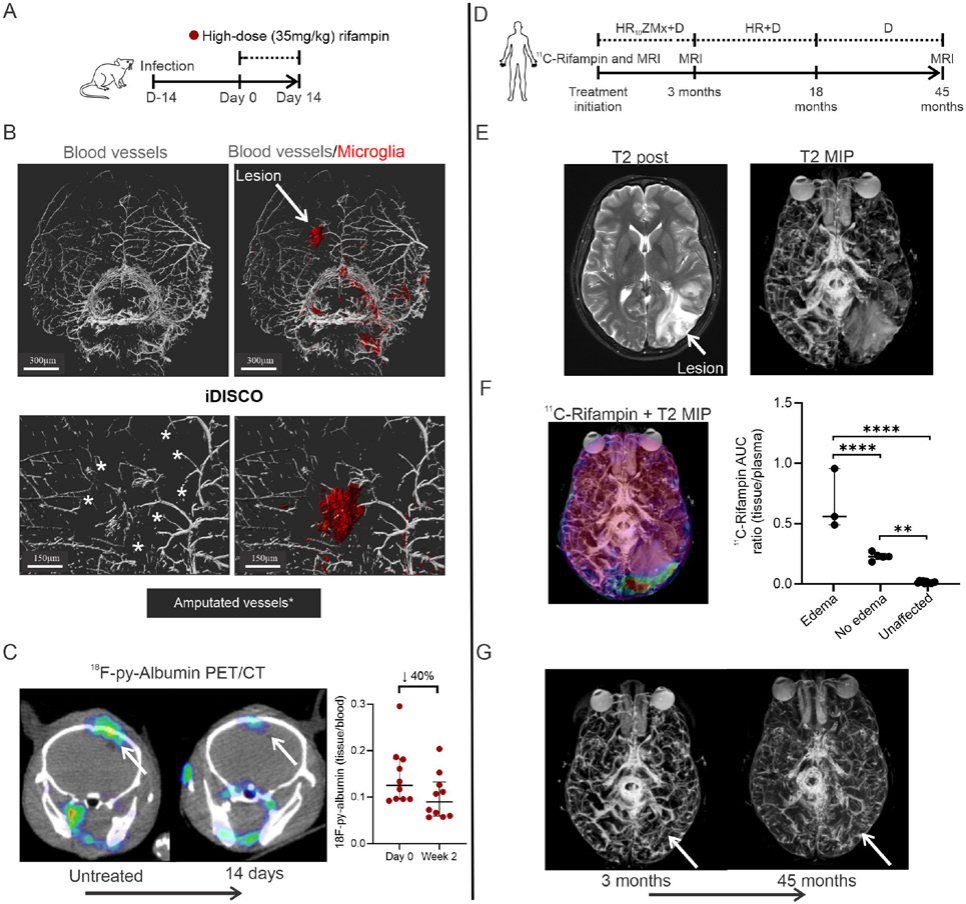

Figure 4. Changes in vascular leakage and rifampin penetration during TB treatment. (A) Experimental scheme in mice. (B) Whole-brain immunolabeling (iDISCO) of a representative M. tuberculosis-infected mouse prior to treatment initiation. Images show immunolabeling of α-smooth muscle actin in grey and microglia activation in red (Iba-1). Asterix represents the areas of vascular amputation. (C) Coronal 18F-py-Albumin PET/CT and quantification (tissue to plasma ratio) in untreated and 2 weeks-treated mice (n = 10 animals at each time-point). (D) Serial imaging in a patient with TB meningitis. (E) Transverse T2 post-contrast section and maximal intensity projection (MIP) showing vasogenic edema. (F) Co-registered T2 post-contrast MIP with transverse 11C-rifampin area under the curve (AUC) heat-map, and 11C-rifampin tissue to plasma ratio quantification of the areas with and without vasogenic edema, and unaffected brain. (G) T2 post-contrast MIP at 3 and 45 months after treatment initiation. The patient with TB meningitis was recruited as part of a 11C-rifampin PET research study performed under the U.S. FDA Radioactive Drug Research Committee program for investigational drugs (Tucker et al. Sci Transl Med. 2018; Ordonez et al. Nat Med. 2020). Human studies were approved by the Johns Hopkins University Institutional Review Board Committee. M = moxifloxacin. PET data is represented as median ± IQ. *P < 0.05, **P < 0.01 and ***P < 0.001 calculated using a two-way ANOVA test.

**Conclusion:**

Our cross-species findings suggest that an intensified high-dose rifampin regimen is more efficacious than the standard treatment for TB meningitis without an increase in neuroinflammation. Vascular leak decreases during TB treatment and may account for decreases in rifampin permeability over time. These studies have important implications for antimicrobial development for TB meningitis.

**Disclosures:**

**Charles A. Peloquin, Pharm.D.**, Nothing to disclose **Alvaro A. Ordonez, MD**, **Cubresa** (Consultant)**Fujirebio Diagnostics** (Research Grant or Support) **Sanjay K. Jain, MD**, **Fujirebio Diagnostics, Inc., USA** (Research Grant or Support)**Novobiotic LLC, USA** (Research Grant or Support)**T3 Pharma, Switzerland** (Research Grant or Support) **Sanjay K. Jain, MD**, Fujirebio Diagnostics, Inc., USA (Individual(s) Involved: Self): Research Grant or Support; Novobiotic LLC, USA (Individual(s) Involved: Self): Research Grant or Support; T3 Pharma, Switzerland (Individual(s) Involved: Self): Research Grant or Support

